# Design and performance study of grease spool-less relief valve

**DOI:** 10.1038/s41598-022-20871-8

**Published:** 2022-10-03

**Authors:** Guoqing Gong, Youmin Wang, Bin Zhou

**Affiliations:** College of Mechanical Engineering, Anhui Polytechnic University, Wuhu, 241000 Anhui China

**Keywords:** Mechanical engineering, Materials for devices

## Abstract

The high resistance characteristic of the grease delivery process makes the centralized lubrication system easy to cause pipeline blockage during the grease supply process. The main objective of the present article is to design a non-spool relief valve device based on the round pipe. The plug shape characteristics of the grease flow in the tube are established by combining the hydrodynamic equation and the grease rheological model. The mathematical model, whose relationship between velocity and flow is derived, is established with the core pipe of the grease spool-less relief valve of flow resistance, so the factors affecting grease pipe flow are obtained. The numerical simulation method is used to simulate the lipid overflow process of the simplified model with different influence parameters. Tecplot 360 EX 2015 R1 is used as a post-processing software to derive velocity and pressure clouds for grease flow in a circular tube, to investigate the factors influencing the relief pressure and relief capacity of the relief valve, to derive general rules for grease relief pressure, grease relief capacity and grease flow pattern distribution, and to establish an evaluation model for the relief pressure and relief capacity of the relief valve. The performance research test platform of the grease spool-less relief valve is built, and NLGI1 lithium grease is selected to carry out the experimental study on the performance of the grease relief valve under different influence parameters. The safety, stability, and feasibility of the overflow valve working with the pumping system are verified.

## Introduction

The grease transported by the centralized lubrication system is different from the hydraulic medium conveyed by the ordinary hydraulic system. The grease shows a high consistency and is easy to clog in narrow gap flow. On the other hand, the standard relief valve has a compact structure, exquisite internal spool structure, narrow flow channel, and abrupt cross-section. It is easy to block if used in the centralized lubrication system. It is of practical significance and value to design a grease spool-free relief valve device to realize overflow protection according to grease characteristics with moderate consistency.

Jingjing Mao et al.^1^ determined the rheological parameters of five kinds of compound lithium grease. The rheological parameters and microstructure of five kinds of greases were compared. The results show that understanding the rheological properties of lubricating grease may be helpful to reflect the change in thickener structure. The flow transition index characterizes the fracture behaviour of the internal structure of the grease; the higher the index, the better the soap fibre structure of the grease, and the damping factor shifts from the medium range to the lower value resulting in the brittleness of the sample. At a constant shear rate, the soap fibre structure of composite lithium grease is compact and uniform, and the decrease rate of apparent viscosity is low. Porfiryev Yaroslav et al.^2^ studied the effects of the properties and composition of the dispersion medium on the physical and chemical properties of low-temperature grease (LTG) thickened with lithium stearate soap, and found the possibility of expanding the working temperature range and improving the antiwear performance of low-temperature grease by combining low pour point mineral oil with high index hydrotreating oil. Delgado et al.^3^ have obtained practical steady-state flow curves from different rheological tests and schemes of five kinds of greases, which contain quite different thickeners, namely aluminium complex, lithium complex and calcium complex soap and polyurea. The experimental results show that because of the strong time dependence and obvious yield behaviour in a wide range of shear rates, the flow such as shear band and fracture is unstable, and it is difficult for these colloidal suspensions to achieve the steady flow condition. In order to better understand these phenomena, the transient flow experiments under constant shear rate, and the creep tests under constant shear stress were carried out using controlled strain and controlled stress rheometer, respectively. The main purpose of this work is to study the steady flow behaviour of grease and analyze how the microstructure characteristics affect the yield flow behaviour. Zakani Behzad et al.^4^ investigated the difference in rheological characterization of fumed silica grease under controlled stress and strain modes. The results of steady-state viscosity measurement in the controlled strain mode reveal the non-monotonic temperature-dependent behaviour. Due to the decrease in stick–slip phenomenon and elastic deformation, the controlled stress and controlled strain rheology are in good agreement with those of high stress and shear rate. Norifumi Miyanaga et al.^5^ described the influence of the flow characteristics of lithium soap grease on the torque of small ball bearings. Cross stress is obtained for grease, the shear stress at Grain G. Then, the bearing torque of the three greases used as lubricants is measured. As a result, the grease with higher cross stress shows lower bearing torque, regardless of its higher apparent viscosity. Paszkowski et al.^6^ studied the regeneration process of micro-morphology of grease under external force by an infrared spectrometer (FTIR). He found that the regenerative recovery state of the grease micromorphology was best maintained in the 0–4 h range and that its rheological properties were also recovered. Westerberg et al.^7^ established a theoretical model of grease flow in rectangular pipes based on the Hmurb rheological model and carried out an experimental analysis to explore the changing law of rheological properties.

First of all, this paper puts forward the grease spool-less relief valve design and designs a kind of spool-less relief valve device with the circular tube as the core prototype. Based on the fundamental equation of hydrodynamics, the mathematical model of flow resistance in the core pipe of the grease spool-less relief valve is established, and the mathematical model of velocity and flow distribution of grease in the pipeline is deduced. Then the overflow performance of the grease spool-less relief valve is studied by Fluent15.0. The relationship between pressure and velocity of overflow unit under different parameters is obtained using the method of numerical fitting. The distribution law is obtained between pipe diameter, pipe length, grease velocity, and pressure. The action mechanism of the grease spool-less relief valve is revealed. Finally, the performance test-bed of the grease spool-less relief valve is set up to analyze the relief valve's output pressure and flow stability and its working efficiency with the pump. Verify the safety stability and feasibility of this relief valve working with the pumping system.

## Design of non-spool relief valve for lubricating grease

**Figure 1:**
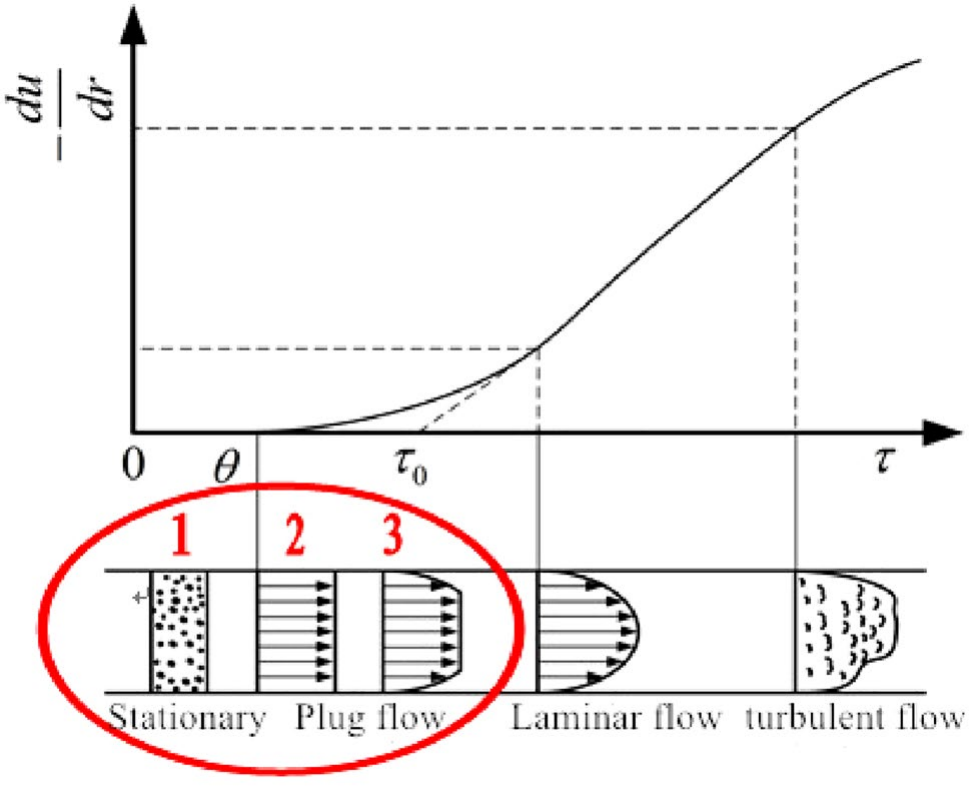
Corresponding relationship between flow pattern distribution and shear rate of grease pipe.

Through the test and analysis of the shear rheological properties of NLGI1 lithium grease^8^, the results show that the shear rate strongly influences the flow pattern of grease. To accurately establish the theoretical mathematical model of spool-free relief valve pipe flow, Fig. 1 shows the corresponding relationship between grease pipe flow pattern distribution and shear rate. The part of the red ring is the evolution process of grease flow in the pipeline. The shear stress formed by applying a lower shear rate in the pipeline can not reach the yield structure strength of the grease itself and will eventually remain in the static non-flow state of the first stage. With the continuous increase of the shear rate applied to the grease, the corresponding shear stress increases synchronously; however, the distribution of the shear rate is relatively low in other areas except for the pipe wall, and the corresponding shear stress still fails to break through the yield structure strength limit of the grease itself, but it is affected by the formation of a higher shear rate at the pipe wall, so it shows a piston-like flow pattern shown in stage 2 in the pipeline. With the continuous increase of the shear rate, the shear stress of the grease exceeds the limit of its yield structure strength near the pipe wall, so there is still a stage 2 piston flow in the central area of the tube, which is also called the flow core. Finally, it presents the plug flow state of the third stage.

### Structural design scheme

**Figure 2:**
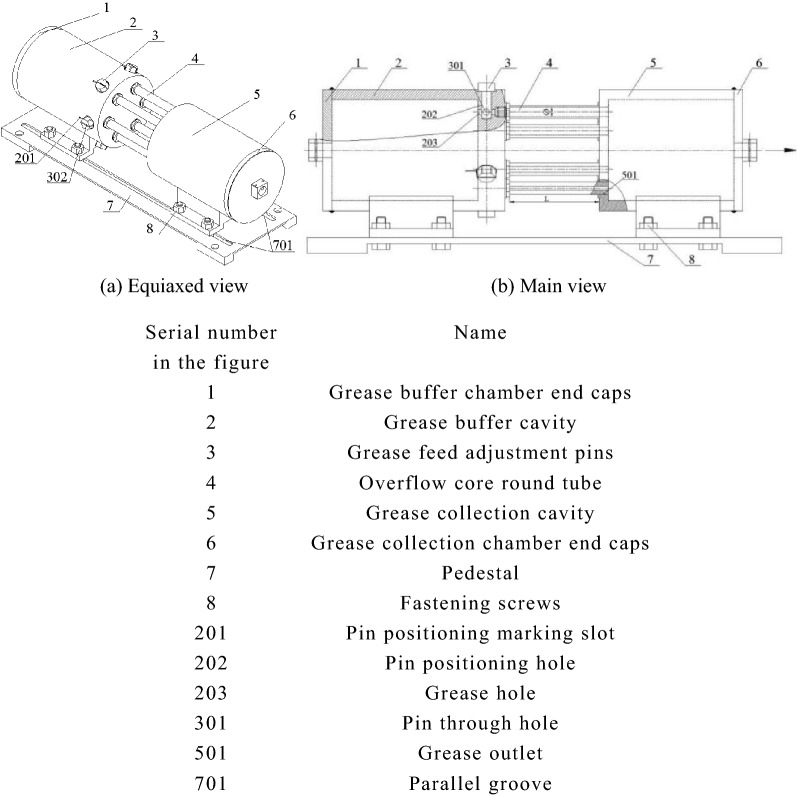
Structure diagram of grease spool-less relief valve.

The core of the working principle of the grease spool-less relief valve is to rely on the resistance of grease in the pipe as the overflow pressure of the valve. Therefore, the core component of the grease overflow device is composed of several round pipes with different diameters, which can meet the requirements of different overflow capacities. When the pipeline of the pumped centralized lubrication system is not smooth, resulting in high pressure, the grease spool-free relief valve begins to work. As shown in Fig. 2: the grease flows into the grease buffer cavity through the inlet of the end cover of the grease buffer chamber. After full filling, the pressure in the cavity rises enough to overcome the shear yield stress of the grease and enters the core tube of the relief valve through the grease outlet hole at the other end of the cavity. There are five core pipes, and the flow of grease in the pipe with variable diameter and length produces varying degrees of resistance. The different overflow capacity of the spool-less relief valve can be realized, which needs to be realized by controlling the five fat intake adjusting pins corresponding to the circular pipe on the grease buffer cavity. A word slot is engraved on the hexagonal nut of the pin, and a circular pin through hole is opened on the pin corresponding to the grease hole. When it is necessary to realize the different overflow capacity of the spool-less relief valve, only by adjusting the word slot on the pin nut and the marked groove on the buffer cavity are perpendicular to each other the grease can flow through the round pipe through the pipe resistance pressure drop, and flow into the grease outlet at one end of the grease collecting cavity to realize the pipeline overflow. Complete the overflow protection of the centralized lubrication system of grease^9^. At the same time, to increase the working stability of the grease spool-less relief valve, the main valve body is fixed with the base, the valve body is connected with the fastening screw to support the ear plate, and the upper part of the ear plate is closely welded with the two cavities. There is a parallel slot on the base, which can adjust the connection distance between the entrance of the grease buffer cavity and the main pumping channel to achieve the best use.

### Design theory of grease spool-less relief valve

The core of the overflow unit of the grease spool-less relief valve is a circular pipe, and its overflow pressure comes from the flow resistance of grease pipe flow. Therefore, the theoretical model of pipe flow of grease is the core of the grease spool-less relief valve design. The analysis of the flow characteristics of grease in the pipe of grease spool-less relief valve relief unit needs to be carried out by combining the three basic conservation laws (1), (2) and (3).

1 $$ {\text{Continuity equation}}{:}\;\frac{\partial \rho }{{\partial {\text{t}}}} + \nabla \cdot \left( {\rho \overrightarrow {v} } \right) = 0 $$

2 $$ {\text{Equation of motion}}{:}\;\rho \frac{Dv}{{Dt}} = - \nabla p + [\nabla \cdot \overrightarrow {\sigma } ] + \rho \cdot \overrightarrow {g} $$

3 $$ {\text{Energy equation}}{:}\;\rho c_{v} \frac{DT}{{Dt}} = - \nabla \cdot \overrightarrow {q} + \sigma :\nabla \overrightarrow {v} $$

“$\overrightarrow {v} , \, \overrightarrow {\sigma } , \, \overrightarrow {g} , \, \overrightarrow {q}$” all are vectors.

**Figure 3:**
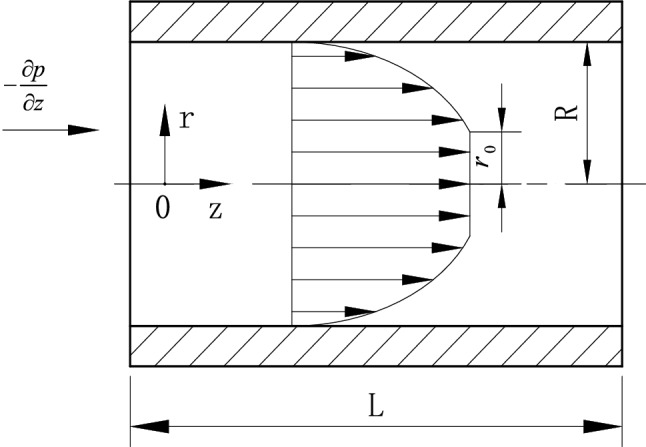
Flow pattern diagram of grease.

Based on the design idea of the plug flow shape of grease, the mathematical model of grease flow in the pipeline, as shown in Fig. 3, is established. Where the axial flow direction of the grease along the pipeline is indicated by z,$\partial p/\partial z$ represents the pressure gradient formed by the grease flowing along the z-direction in the pipeline because the pressure gradient is a vector, and the pressure formed by the grease flowing along the z-direction decreases gradually, so the pressure gradient should be negative. In addition, the grease pipeline flow is a plug flow state with a flow core zone, the flow core is R0, the inner ring radius of the pipeline is R, and the pipe length is L, based on the parameters given above theoretical model is further established.

### Establishment of theoretical model of grease spool-less relief valve

According to the mathematical model of grease flow in the pipeline, the three conservation laws in fluid medium flow are also applicable to grease flow. Because the flow direction of grease is γ direction, θ direction and z direction respectively, it is necessary to decompose v, σ, g q in formulas (1), (2), (3) according to three major conservation laws^10^, such as formula (4), (5), (6), (7), (8).

4 $$ {\text{Continuity equation}}{:}\;\frac{\partial \rho }{{\partial t}} + \left[ {\frac{{\partial \left( {r\rho v_{r} } \right)}}{r\partial r} + \frac{{\partial \left( {\rho v_{\theta } } \right)}}{r\partial \theta } + \frac{{\partial \left( {\rho vz} \right)}}{\partial z}} \right] = 0 $$

Momentum equation:

5 $$ \begin{aligned} & \gamma {\text{ direction}}{:}\;\rho \left( {\frac{{\partial v_{r} }}{\partial t} + v_{r} \frac{{\partial v_{r} }}{\partial r} + \frac{{v_{\theta } }}{r}\frac{{\partial v_{r} }}{\partial \theta } - \frac{{v_{\theta }^{2} }}{r} + v_{z} \frac{{\partial v_{r} }}{\partial z}} \right) \hfill \\ & \quad = - \frac{\partial p}{{\partial r}} + \left( {\frac{1}{r} \cdot \frac{\partial }{\partial r}\left( {r\sigma_{rr} } \right) + \frac{1}{r} \cdot \frac{{\partial \sigma_{r\theta } }}{\partial \theta } - \frac{{\sigma_{\theta \theta } }}{r} + \frac{{\partial \sigma_{rz} }}{\partial z}} \right) + \rho g_{r} \hfill \\ \end{aligned} $$

6 $$ \begin{aligned} & \theta {\text{ direction}}{:}\; \rho \left( {\frac{{\partial v_{\theta } }}{\partial t} + v_{r} \frac{{\partial v_{\theta } }}{\partial r} + \frac{{v_{\theta } }}{r}\frac{{\partial v_{\theta } }}{\partial \theta } - \frac{{v_{r} v_{\theta } }}{r} + v_{z} \frac{{\partial v_{\theta } }}{\partial z}} \right) \hfill \\ & \quad = - \frac{1}{r} \cdot \frac{\partial p}{{\partial r}} + \left( {\frac{1}{{r^{2} }} \cdot \frac{\partial }{\partial r}\left( {r^{2} \sigma_{r\theta } } \right) + \frac{1}{r} \cdot \frac{{\partial \sigma_{\theta \theta } }}{\partial \theta } + \frac{{\partial \sigma_{\theta z} }}{\partial z}} \right) + \rho g_{\theta } \hfill \\ \end{aligned} $$

7 $$ \begin{aligned} & {\text{z direction}}{:}\;\rho \left( {\frac{{\partial v_{z} }}{\partial t} + v_{r} \frac{{\partial v_{z} }}{\partial r} + \frac{{v_{\theta } }}{r}\frac{{\partial v_{z} }}{\partial \theta } + v_{z} \frac{{\partial v_{z} }}{\partial z}} \right) \hfill \\ & \quad = - \frac{\partial p}{{\partial r}} + \left( {\frac{1}{r} \cdot \frac{\partial }{\partial r}\left( {r\sigma_{rz} } \right) + \frac{1}{r} \cdot \frac{{\partial \sigma_{\theta z} }}{\partial \theta } + \frac{{\partial \sigma_{zz} }}{\partial z}} \right) + \rho g_{z} \hfill \\ \end{aligned} $$

Energy equation:

8 $$ \begin{aligned} \rho c_{v} \frac{DT}{{Dt}} & = - \left( {\frac{1}{r}\frac{\partial }{\partial r}\left( {rq_{r} } \right) + \frac{1}{r}\frac{{\partial q_{\theta } }}{\partial \theta } + \frac{{\partial q_{z} }}{\partial z}} \right) - T\left( {\frac{\partial p}{{\partial T}}} \right)_{\rho } \cdot \left( {\frac{1}{r}\frac{\partial }{\partial r}\left( {rv_{r} } \right) + \frac{1}{r}\frac{{\partial v_{\theta } }}{\partial \theta } + \frac{{\partial v_{z} }}{\partial z}} \right) \hfill \\ & \quad + \left[ {\sigma_{rr} \frac{{\partial v_{r} }}{\partial r} + \sigma_{\theta \theta } \frac{1}{r}\left( {\frac{{\partial v_{\theta } }}{\partial \theta } + v_{r} } \right) + \sigma_{zz} \frac{{\partial v_{z} }}{\partial z}} \right] \hfill \\ & \quad + \left[ {\sigma_{r\theta } \left( {r\frac{\partial }{\partial r}\left( {\frac{{v_{\theta } }}{r}} \right) + \frac{1}{r}\frac{{\partial v_{r} }}{\partial \theta }} \right)} \right] + \left[ {\sigma_{rz} \left( {\frac{{\partial v_{z} }}{\partial r} + \frac{{\partial v_{r} }}{\partial z}} \right)} \right] + \left[ {\sigma_{\theta z} \left( {\frac{1}{r}\frac{{\partial v_{z} }}{\partial \theta } + \frac{{\partial v_{\theta } }}{\partial z}} \right)} \right] \hfill \\ \end{aligned} $$

### Solution of theoretical model of lubricating grease spool-less relief valve

By properly simplifying the Eqs. (4), (5), (6) and (7), the basic equations of grease circular pipe flow are obtained as follows:

9 $$ {\text{Continuity equation}}{:}\;\frac{{\partial v_{z} }}{{\partial {\text{z}}}} = 0 $$

10 $$ {\text{Momentum equation}}{:}\;\frac{\partial p}{{\partial {\text{z}}}} = \frac{1}{r} \cdot \frac{\partial }{\partial r}\left( {r\sigma_{rz} } \right) $$

Constitutive equation of Hmurb rheological model:

11 $${\upsigma }_{\mathrm{rz}}=\uptau ={\uptau }_{0}+\mathrm{k}{(\dot{\gamma })}^{\mathrm{n}}=\uptau 0+\mathrm{k}{\left(\frac{\partial {\mathrm{v}}_{\mathrm{z}}}{\partial \mathrm{r}}\right)}^{\mathrm{n}}$$

The simplified theoretical model of grease spool-less relief valve is solved by combining formulas (9), (10) and (11), and the corresponding velocity vz equations are obtained as follows:

12 $$ {\text{v}}_{{\text{z}}}  = \left\{ {\begin{array}{ll}    { - \left( {\frac{1}{{2k}}} \right)^{{\frac{1}{n}}} \left( {\frac{n}{{n + 1}}} \right)R^{{\frac{{n + 1}}{n}}} \left( {\frac{{\partial p}}{{\partial z}}} \right)^{{ - 1}} \left[ {\left( {\frac{{\partial p}}{{\partial z}} - \frac{{2\tau _{0} }}{R}} \right)^{{\frac{{n + 1}}{n}}}  - \left( {\frac{{\partial p}}{{\partial z}}\frac{r}{R} - \frac{{2\tau _{0} }}{R}} \right)^{{\frac{{n + 1}}{n}}} } \right]   \left( {r_{0}  < r \le R} \right)}  \\    { - \left( {\frac{1}{{2k}}} \right)^{{\frac{1}{n}}} \left( {\frac{n}{{n + 1}}} \right)R^{{\frac{{n + 1}}{n}}} \left( {\frac{{\partial p}}{{\partial z}}} \right)^{{ - 1}} \left( {\frac{{\partial p}}{{\partial z}} - \frac{{2\tau _{0} }}{R}} \right)^{{\frac{{n + 1}}{n}}} {\text{                                  }} \left( {{\text{0}} \le r \le r_{0} } \right)}  \\   \end{array} } \right. $$

According to formula (12), the velocity equation of grease flow in the simplified model of spool-less relief valve well describes the flow core region and viscous flow region of grease flow in the pipeline.To more clearly define the flow distribution of grease in the simplified model of the spool-less relief valve, the integral solution of Eq. (12) is carried out to obtain the flow rate of grease in the simplified model of the spool-less relief valve.

13 $$ q = \int_{0}^{R} {v_{z} 2\pi r} dr = \pi r_{0}^{2} v_{z} \left( {r_{0} } \right) + \int_{{r_{0} }}^{R} {v_{z} 2\pi r} dr $$

If the length of grease spool-less relief valve is L, the pressure is:

14 $$ p = L \cdot \partial p/\partial z $$

The pressure of the spool-free relief valve is:

To realize the overflow effect, the flow rate of the circular pipe flow must be close to 0, and the pressure difference between the two ends of the pipe is the overflow pressure of the grease relief valve, that is:

15 $$ \mathop {\lim }\limits_{q \to 0} f(q,r) = P(const) $$

Equations (13) and (15) show that the pressure equation for grease flow in the simplified model of a spool-less relief valve is a physical quantity influenced by multiple factors. Therefore, it is necessary to analyze further the theoretical model of grease flow in a spool-less relief valve.

According to the above analysis results, the flow rate and grease pressure in the simplified spool-less relief valve model are complex physical quantities affected by many factors. The factors that affect the flow rate and flow rate of grease in the simplified model of a spool-less relief valve can be divided into two categories: rheological parameters ($\tau 0$,*k*,*n*) and pipeline structure parameters (*R, L*)^11^. The nature of the grease itself determines the former. The pipe diameter is the structural parameter of the spool-less relief valve, which is determined by structural design.

## Grease valve overflow valve grease spool-free overflow threshold

The grease in the spool-less relief valve is a three-dimensional flow with a circular tube as the core model. In order to facilitate the smooth development of the numerical simulation, the model is simplified in the following aspects: (1) the heat conduction of the medium in the transportation process has little effect on the grease flow; (2) the wall slip phenomenon caused by the grease is ignored; (3) all parts of the valve body are well sealed in the simulation process. (4) the grease keeps steady flow in the spool-less relief valve, and (5) the grease is an incompressible medium.

### Setting of numerical simulation model and boundary conditions

**Figure 4:**
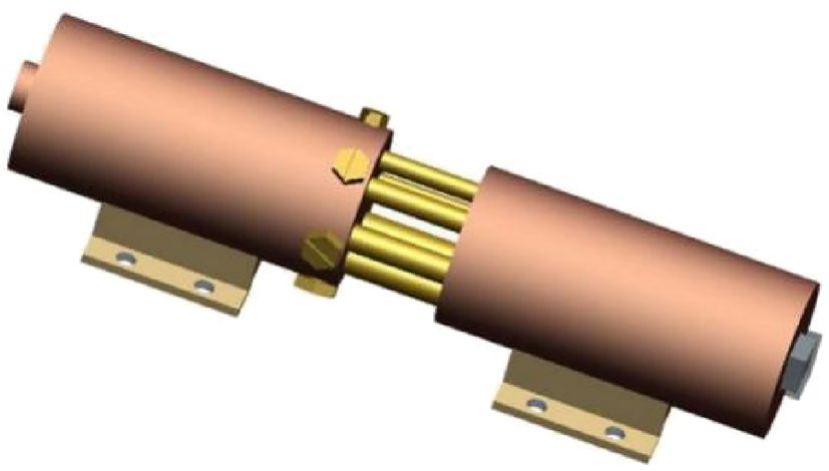
Three-dimensional geometric model of spool-less relief valve.

The structure of the spool-free relief valve is modelled by using the professional 3D drawing software Pro/Engineer5.0. The geometric model is shown in Fig. 4.

The rated flow rate of the grease pumped by the lubrication system is set to 30 ml/min, and its velocity corresponds to 0.00637 m/s. In the numerical simulation of the grease pipe flow, the inlet in the boundary condition is set as the grease velocity, and the outlet condition is set as the pressure outlet. Considering that there is almost no pressure from the grease overflow to the outlet, the outlet pressure is set to 0Mpa, which can effectively improve the accuracy of numerical simulation^12^. The basic parameters of the specific boundary conditions are shown in Table 1.

**Table 1:** Basic parameters of boundary conditions.

Calculation condition	Parameters
Density	900 kg/m^3^
Consistency Index, k	286.4 kg/m s^−1^
Power-Law Index, n	0.43
Yield stress threshold	542 Pa
Critical shear rate	0.0631 s^−1^
Inlet	0.00637 m/s
Outlet	0 Mpa
Upwind	Second Order Upwind
Method	Simple

### Parameter setting of numerical simulation model grouping

From the mathematical model of the grease flow pattern established in Eqs. (12) and (13), we know that the influencing factors of grease flow include the flow rate and the size of the core circular tube diameter. In this paper, by setting five groups of pipe diameters (D = 2 mm, 4 mm, 6 mm, 8 mm and 10 mm) and three pipe lengths (L = 50 mm, 100 mm, and 150 mm), and carrying out 75 numerical analyses of grease core pipe flow under five inlet flow modes, the flow performance of grease spool-less relief valve is discussed in detail, and the influence of key factors on the performance of the relief valve is analyzed. Further clear selection of appropriate parameters for the construction of the testbed to prepare for the test. Table 2 shows the specific simulation analysis parameters of each group.

**Table 2:** Specific simulation analysis parameters of each group.

Number of groups	Pipe diameter (mm)	Pipe chief (mm)	Inlet traffic (ml/min)	Remarks
5	2/4/6/8/10	50	10	Minimum flow
			15	
		100	20	
			25	
		150	30	Maximum flow

### Numerical simulation results and analysis of overflow pressure

The inlet flow rate is set to explore the influence factors of pipe diameter and pipe length on the flow grease in the circular tube. The specific numerical simulation is divided into the following cases.

The pipe diameter is 2 mm, the inlet flow is 30 ml, the outlet pressure is default 0Mpa, the numerical simulation is made by Fluent software^13^, and the flow velocity and pressure cloud diagram of grease pipe are derived by Tecplot360EX2015R1 post-processing software.

**Figure 5:**
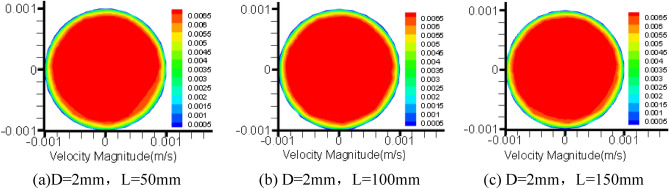
Cloud picture of velocity vector in the middle radial section of three kinds of tube length grease.

Figure 5 shows that the velocity distribution of grease in the radial section of a 2 mm pipe with three pipe lengths is unstable, especially when the grease is close to the pipe wall.

**Figure 6:**
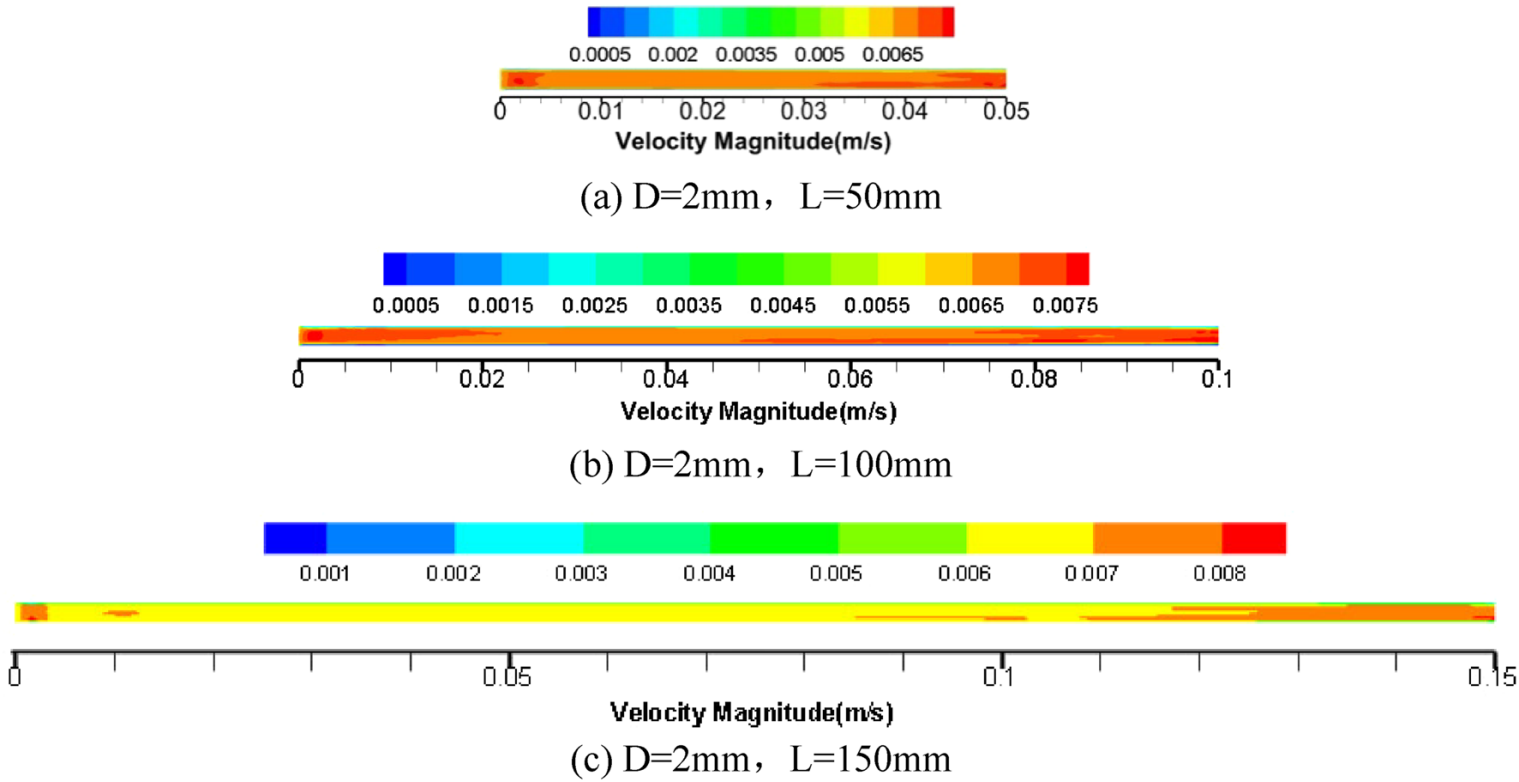
Velocity vector cloud picture of intermediate axial section of three kinds of tube length grease.

Figure 6 shows the velocity cloud diagram of the axial section in the centre of the circular pipe. It is obvious that the velocity of the grease flowing through the middle section of the pipe diameter is relatively stable and uniformly distributed in the pipe. Still, the velocity fluctuates in the inlet and outlet section. At the same time, compared with the middle section of the pipe, the velocity is more significant. The main reason for this phenomenon is that the tube diameter is too small, and the grease needs to overcome the yield stress of the grease itself when it enters the starting section of the circular tube flow^14^, and the unstable pressure leads to flow rate fluctuations.When the grease flows out of the circular tube, due to the very small diameter of the tube, the outflow diameter suddenly changes, the pressure suddenly changes, the loss of tangential stress occurs, and the need to overcome the yield stress of the grease itself causes the velocity field to change rapidly, resulting in unstable flow rate.

**Figure 7:**
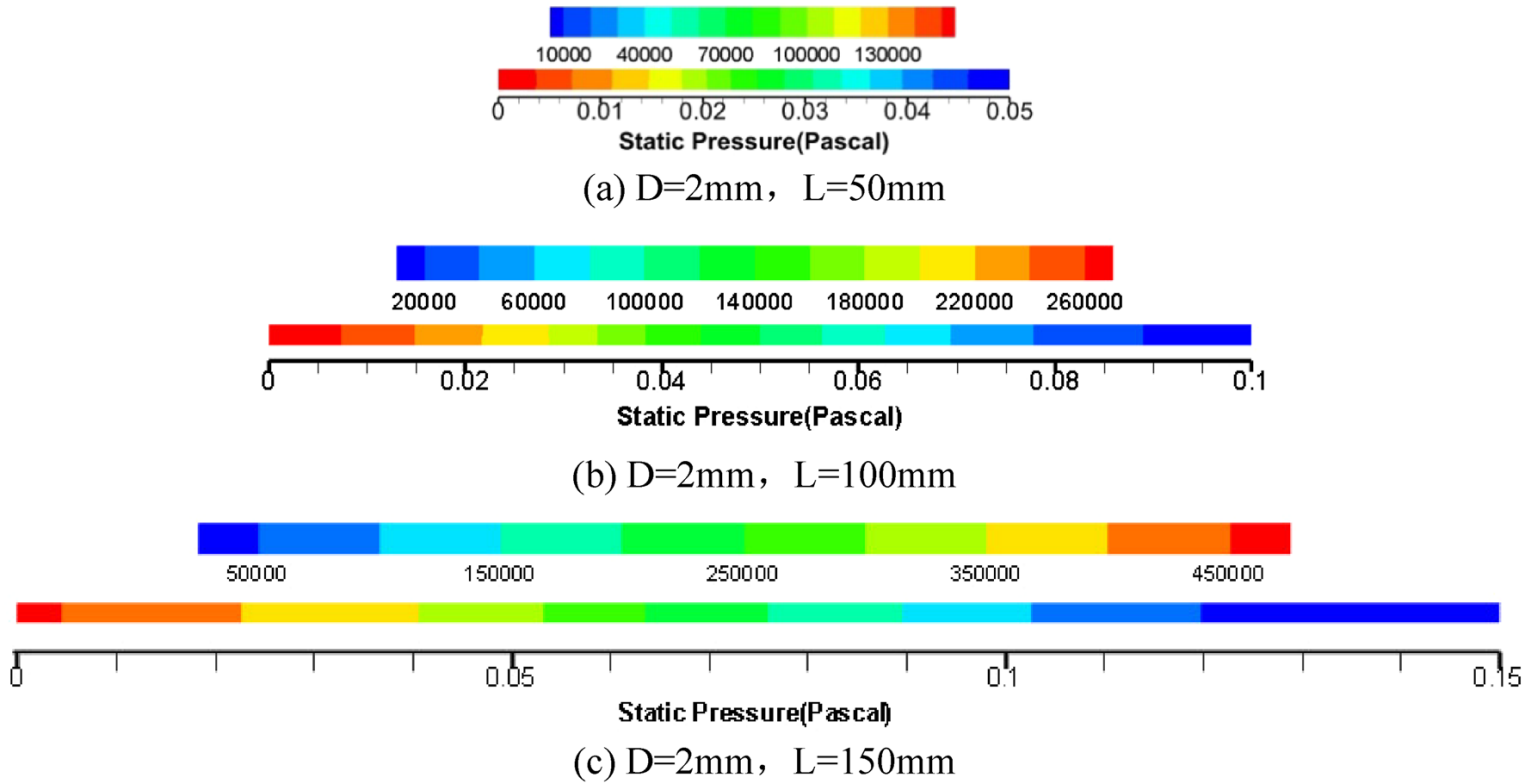
Cloud diagram of axial pressure of grease of three pipe length specifications.

Figure 7 gives the axial pressure cloud of the circular tube, from which it can be analysed that the longer the length of the circular tube, the greater the pressure loss along the diameter of the tube under the condition of certain pipe diameter.

**Figure 8:**
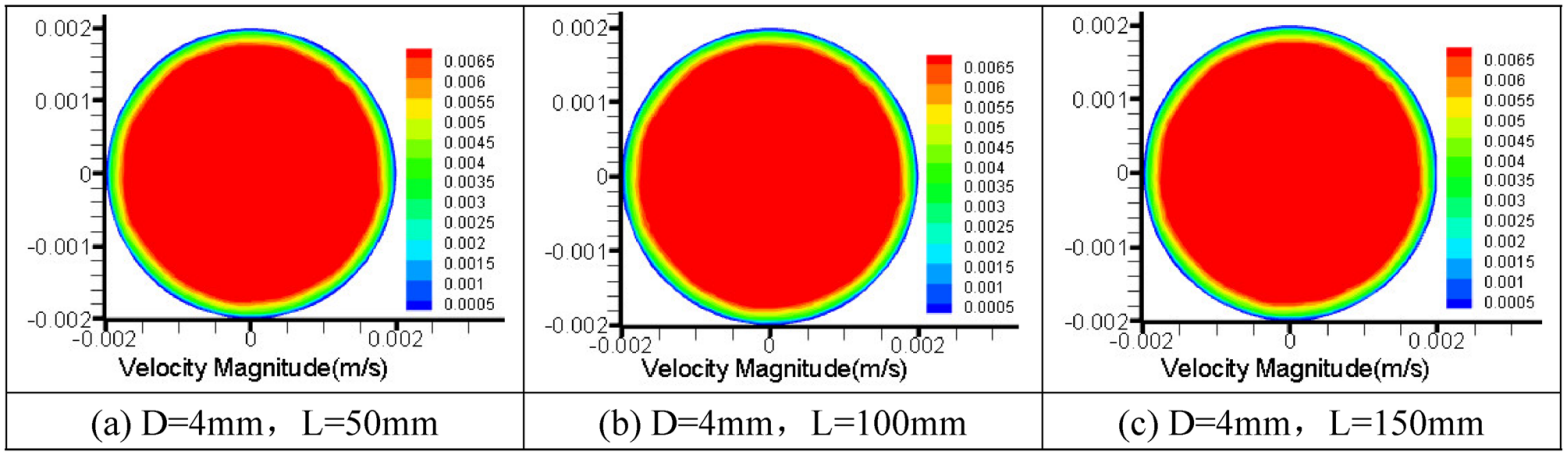
Cloud picture of velocity vector in the middle radial section of three kinds of tube length grease.

**Figure 9:**
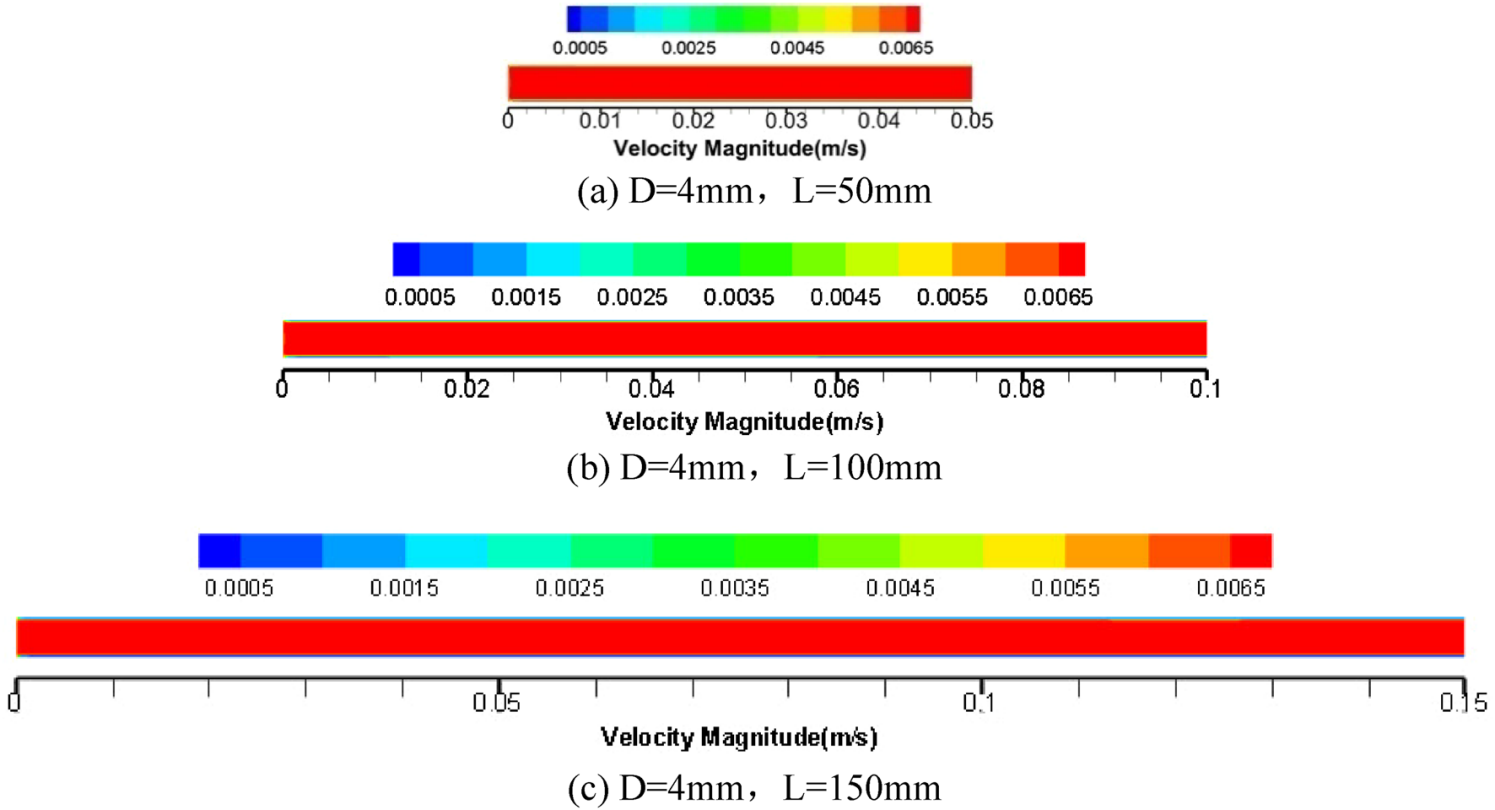
Cloud picture of velocity vector in the central axial section of three kinds of tube length grease.

**Figure 10:**
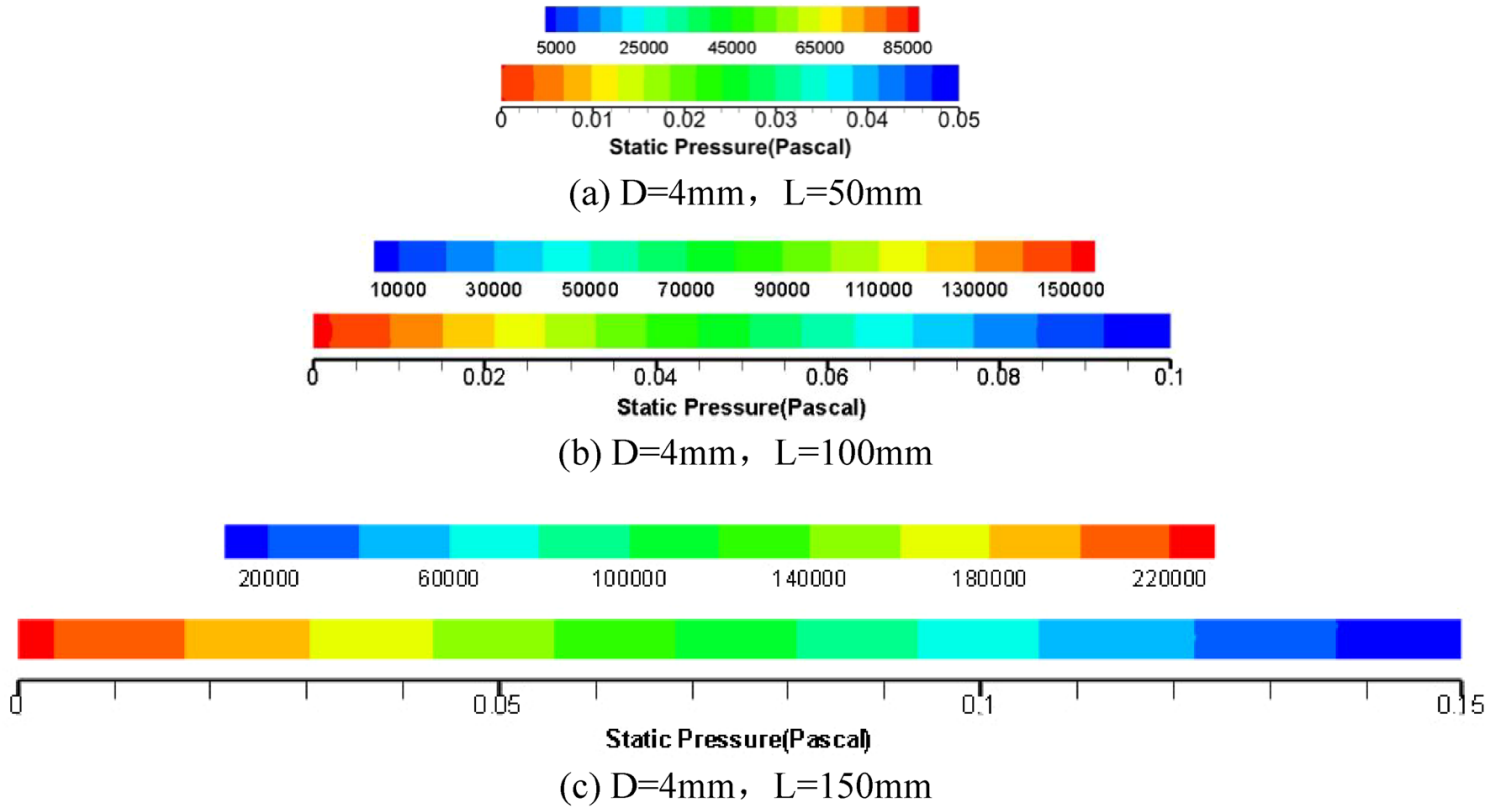
Cloud diagrams of axial pressure of grease of three pipe length specifications.

To further explore the influence of pipe diameter and pipe length on grease flow through the core tube of the relief valve, the numerical simulation results of 4 mm pipe diameter are given as shown in Figs. 8, 9 and 10.

The radial velocity cloud picture 8 of 4 mm pipe diameter grease of three pipe length specifications is relatively lower than that of 2 mm pipe diameter near the pipe wall, and the radial cross-section velocity distribution of grease tends to be stable as a whole.

The radial velocity cloud diagram 9 of the grease shows a stable flow pattern. The velocity distribution of the grease flowing through the three kinds of pipe is relatively stable, the velocity fluctuation in the initial section is not apparent, and the grease is uniformly distributed in the tube. This phenomenon indicates that grease flow in a 4 mm round tube is more stable than flowing through a 2 mm round tube. Therefore, grease flow in a 4 mm tube diameter is relatively ideal.

The axial pressure cloud diagram of grease in a 4 mm diameter pipe is shown in Fig. 10. Compared with a 2 mm pipe, the pressure loss along pipe diameter increases with the increase of pipe length. At the same time, it is concluded that the increase in pipe diameter will lead to a pressure decrease; that is, the overflow pressure of grease spool-less relief valve is related to pipe length and affected by pipe diameter. This conclusion verifies the feasibility of the theoretical derivation of the factors influencing the flow rate Eq. (12) and flow rate Eq. (13) for grease round tubes.

Under several conditions, the various rules of velocity and overflow pressure of grease spool-less relief valve simplified model are analyzed. It is found that the velocity distribution of grease flow in pipe diameters 4 mm, 6 mm, 8 mm and 10 mm shows similar regularity. The essential condition of grease flow is 30 ml/min is the flow rate of grease. Table 3 gives the velocity distribution pattern of the grease pipeline corresponding to each pipe diameter and pipe length in detail.

**Table 3:** Flow rate distribution pattern of grease line for tube diameter and tube length at 30 ml/min.

Pipe diameter (mm)	Status		
Pipe chief (mm):	50	100	150
2	Uneven distribution at both ends	Uneven distribution at both ends	Uneven distribution at both ends
4	Uniform flow distribution	Uniform flow distribution	Uniform flow distribution
6	Uniform flow distribution	Uniform flow distribution	Uniform flow distribution
8	Uniform flow distribution	Uniform flow distribution	Uniform flow distribution
10	Uniform flow distribution	Uniform flow distribution	Uniform flow distribution

**Figure 11:**
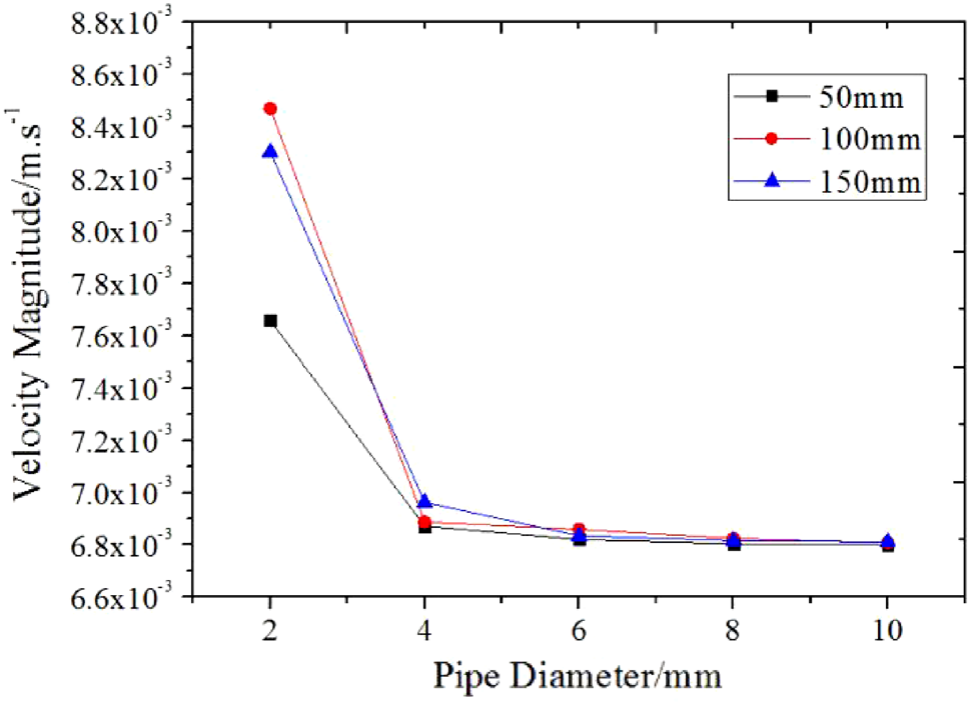
Relationship between grease velocity and pipe diameter.

Based on the analysis of the flow velocity of grease pipe flow in axial and radial sections of five groups of pipe diameters and each group of three kinds of pipe diameters, the results of numerical simulation are summarized, and the relationship between grease velocity and pipe diameter is drawn as shown in Fig. 11.

It can be more clearly analyzed from Fig. 11 that the flow velocity of grease decreases with the increase of pipe diameter. The velocity change tends to be smooth and approximately equivalent in expanding from 4 mm pipe diameter to 10 mm pipe diameter. Combined with the results of five groups of velocity cloud images of numerical simulation, the velocity fluctuation appears obviously at the beginning of the flow of grease in the pipe with a diameter of 2 mm. The tube with a diameter of 4 mm has a certain influence on the flow stability of grease, but the interference fluctuation is not apparent. The steady-state of grease flow in the pipe of 6 mm, 8 mm, and 10 mm is the same; at the same time, the change in pipe length will not affect the flow rate of grease.

**Figure 12:**
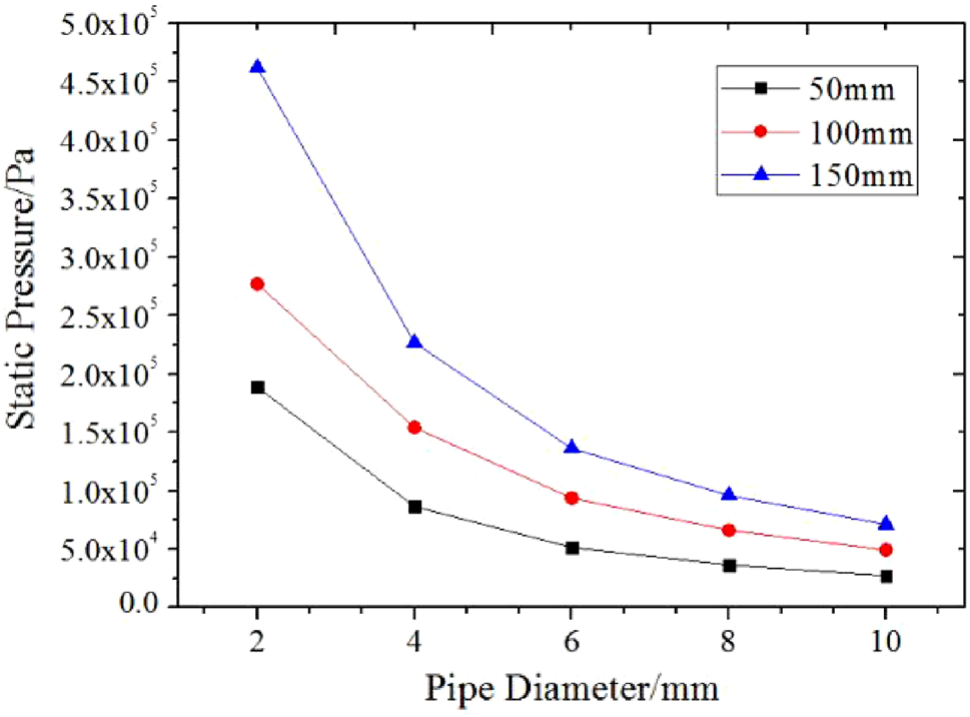
The variation of pipe flow pressure of grease with pipe diameter and pipe length.

Based on the pressure analysis of the axial and radial section of grease pipe flow, the relationship between grease pipe flow pressure and pipe diameter and pipe length is given, as shown in Fig. 12.

Figure 12 shows that the pressure of grease flow along the core tube decreases with the increase of pipe diameter, and the change of pipe diameter pressure of the same size tube increases gradually with the rise of pipe length. Therefore, the pipe with a 2 mm diameter has excellent resistance to grease mm (mm), and the pipe length of 150 mm specification can reach the resistance above 4.5 MPa. Given the inevitable velocity fluctuation of grease flow in the initial 2 mm pipe diameter section, it is bound to cause vibration impact on the spool-free relief valve. General centralized lubrication system equipment can not reach such a high pressure; we can not consider the choice of a 2 mm diameter pipe as the core pipe of the relief valve. The circular tube with a 4 mm diameter can play a strong pipe resistance effect. The grease flow is quite stable, so it is ideal to use the relatively long 4 mm diameter tube to replace the core tube with a 2 mm diameter. The flow of grease under pipe diameters 6 mm, 8 mm, and 10 mm leads to the gradual decrease of pipe diameter pressure, and the pipe length has a far-reaching influence on pipe diameter resistance. So that core round tubes of different diameters can be used to achieve different overflow capacity requirements. Through the analysis of the pipe diameter pressure and flow velocity caused by the pipe diameter and pipe length on the grease pipe flow, it is concluded that under the condition of a particular pipe diameter, the change of the pipe length has no effect on the grease flow velocity, and the internal pipe flow shape of the grease is relatively stable. When the pipe length is fixed, the overflow pressure formed by grease along the core pipe decreases gradually with the increase of pipe diameter, and the downward trend gradually slows down.

From the above grease without spool relief valve along with the core circular tube flow speed and numerical pressure simulation, and then combined with Tables 4 and 5 in each group of pipe length and pipe diameter and adjacent pipe diameter of grease line pressure ratio multiplier relationship can be analyzed to derive specific laws: the pipeline pressure ratio of grease between the adjacent pipe diameter ratios of three groups of pipe lengths is in good agreement, and the pipeline pressure ratio decreases gradually with the increase of pipe diameter. In addition, there is a good correlation between the pressure caused by the flow of grease along the circular tube and the pipe length ratio, and the pressure magnification between each pipe diameter is almost the same when the pipe length ratio is constant, that is to say, the pressure ratio of the adjacent pipe length is not affected by the change of the pipe diameter. And at the same pipe diameter, the pressure ratio of adjacent grease decreases with the increase of pipe length, which indicates that NLGI1 lithium grease maintains an excellent steady flow state in the core tube of the spool-free relief valve^15^.

**Table 4:** Pressure ratio multiplier relationship between each group of tube length and adjacent tube diameter at 30 ml/min.

Pipe chief (mm)	Multiple ratio		
Pipe diameter ratio:	4/6	6/8	8/10
50	1.681	1.411	1.332
100	1.643	1.417	1.341
150	1.663	1.42	1.346

**Table 5:** Pressure ratio multiplier relationship between each group of tube diameter and adjacent tube length at 30 ml/min.

Tube length ratio	Multiple ratio			
Pipe diameter (mm):	4	6	8	10
50/100	1.781	1.822	1.814	1.801
100/150	1.471	1.453	1.45	1.444

### Numerical simulation results and analysis of overflow capacity

To further analyze the influence factors of grease pipe flow, a certain pipe length is set below, and different inlet velocity conditions are given to explore the correlation and variation law of grease overflow flow and overflow pressure in the core pipe of the non-spool relief valve. Because when the centralized grease supply system supplies grease to the equipment, it can usually meet the normal and efficient operation of the equipment under a very low grease flow rate. Based on the grease flow rate provided by the existing equipment in today's society, the inlet flow rate of grease in the core pipe of the spool-less relief valve is set as 10 ml/min, 15 ml/min, 20 ml/min, 25 ml/min and 30 ml/min, and the influence of flow rate on the overflow pressure of the core pipe of the relief valve is analyzed in detail.

**Figure 13:**
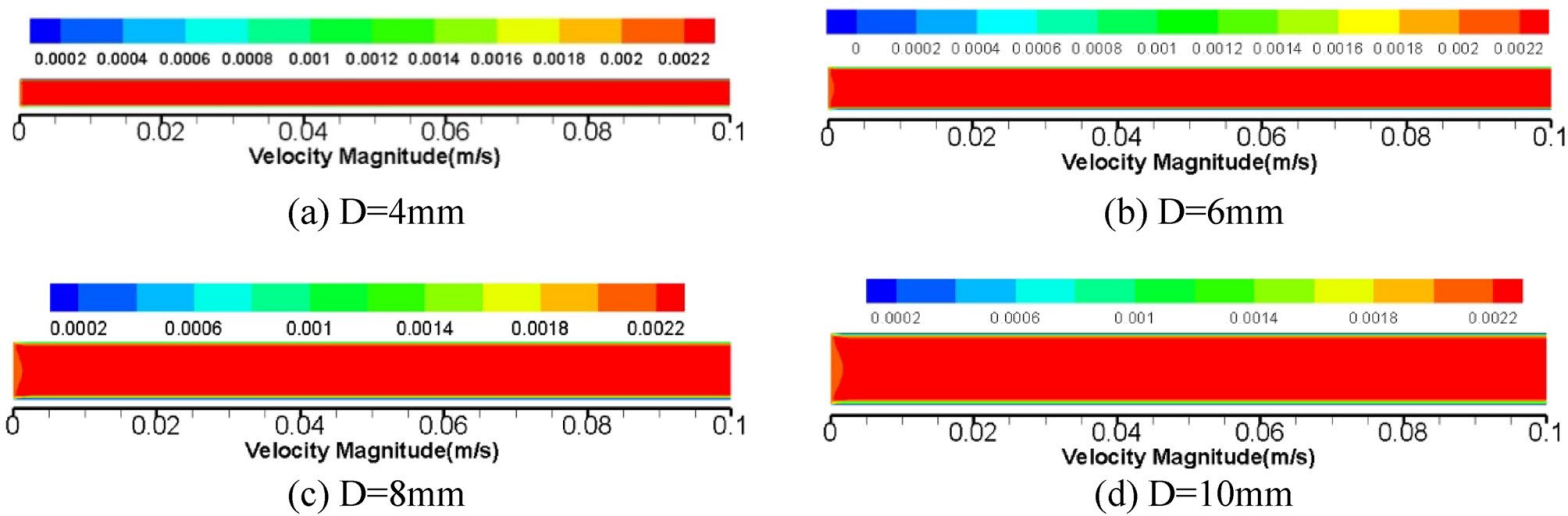
Cloud diagram of velocity distribution of grease pipeline for flow 10 ml/min.

**Figure 14:**
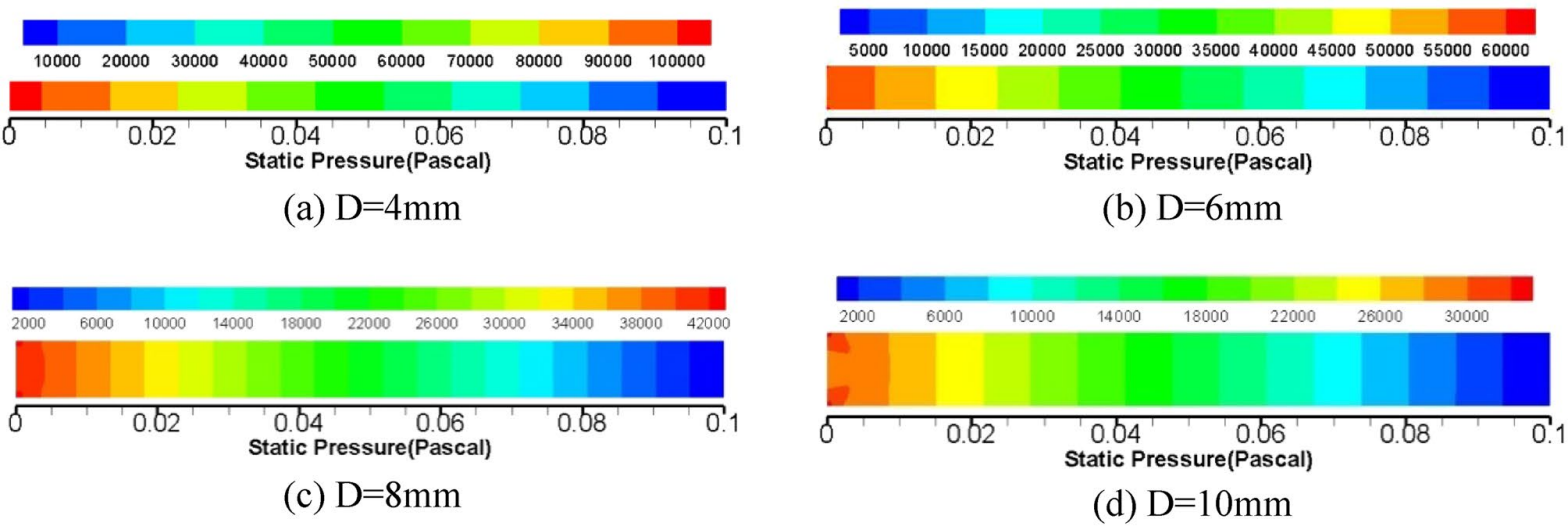
Cloud diagram of flow pressure distribution in grease pipeline of flow 10 ml/min.

Considering the instability of grease flow in the core tube of diameter 2 mm, the following numerical simulation is carried out from the diameter 4 mm tube. L = 100 mm round pipe is selected, and the diameter of the pipe is divided into four groups: 4 mm, 6 mm, 8 mm, and 10 mm. The inlet flow rate is 10 ml/min, and the outlet pressure is set to 0Mpa. The flow velocity of grease in the pipeline is calculated by Fluent15.0 software, as shown in Fig. 13 and the pressure cloud diagram in Fig. 14.

From the cloud chart 13 of the flow rate 10 ml/min grease pipeline, it can be seen that along the axial direction of the pipe diameter, the grease is almost filled with the highest velocity in the whole tube cavity, and the flow distribution is balanced and stable, and the speed near the pipe wall decreases gradually and reaches a static state. In other words, the shear rate of grease is the highest at the pipe wall. In the process of radial extension to the pipe wall with the pipe axis as the center, the grease velocity remains the same, and the shear rate is zero. Therefore, the flow of grease in the pipeline as a whole accords with the morphological characteristics of plug flow.

Figure 14 shows that the mobility of grease keeping flow 10 ml/min shows a certain change law of pipeline pressure. With the increase of the diameter of the core pipe of the relief valve, the pipeline resistance of the tube to the grease tends to decrease, and there is a certain pressure drop relationship between the adjacent diameters, including the pressure value of group d is 1.317 times that of group c, and that of group c is 1.384 times that of group b. The pressure in group b was 1.736 times higher than that in group a. The law of pressure variation between different pipe diameters is analyzed, which is helpful for the experiment to verify the correctness of the numerical simulation results.

The numerical simulation of five groups of different inlet velocities of grease pipeline flow found that the results of cloud images have similar regularities. In order to avoid redundant data cloud images, the numerical simulation results will be presented in the form of Tables 6 and 7.

**Table 6:** Degree of stability of flow pattern of different flow rate grease lines.

Flow (ml/min)	Form			
Pipe diameter (mm):	4	6	8	10
10	Stable distribution	Stable distribution	Stable distribution	Stable distribution
15	Stable distribution	Stable distribution	Stable distribution	Stable distribution
20	Stable distribution	Stable distribution	Stable distribution	Stable distribution
25	Stable distribution	Stable distribution	Stable distribution	Stable distribution
30	Stable distribution	Stable distribution	Stable distribution	Stable distribution

**Table 7:** Grease flow rate and adjacent pipe diameter pressure ratio multiplier relationship.

Flow (ml/min)	Multiple ratio		
Pipe diameter ratio:	6/4	8/6	10/8
10	1.736	1.384	1.317
15	1.732	1.395	1.329
20	1.735	1.391	1.333
25	1.718	1.394	1.342
30	1.643	1.417	1.341

To further explore the variation law of grease flow rate on the resistance effect of grease flow in the core pipe, the numerical simulation results are plotted.

**Figure 15:**
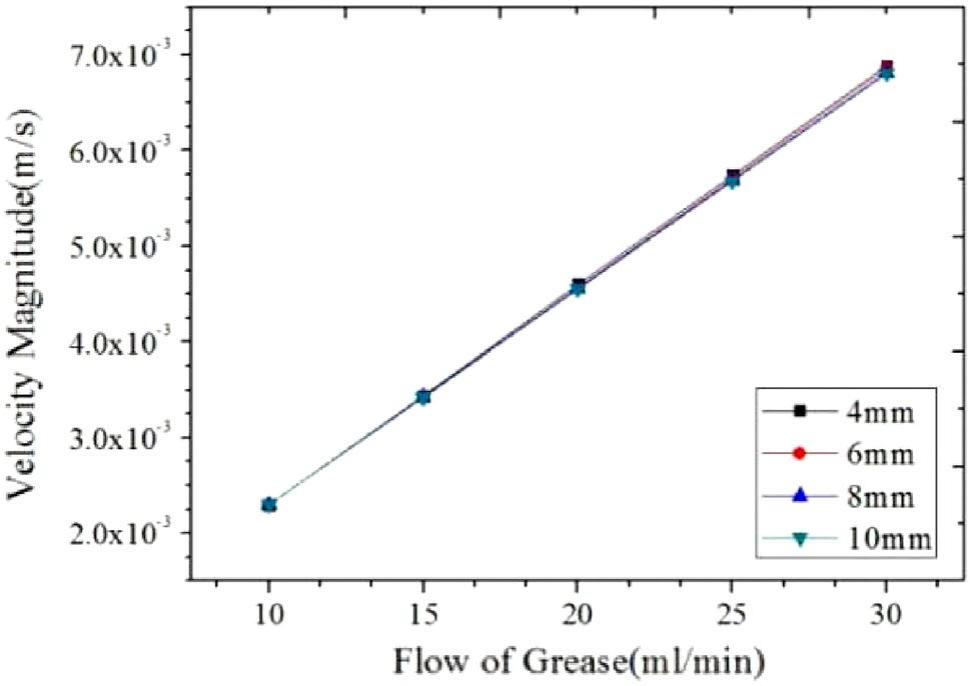
Relationship between flow velocity and flow rate in grease pipeline.

According to the analysis of Fig. 15, under the condition of the set inlet flow rate, the outlet velocity of the grease is the same as the inlet velocity through the action of the core pipe of the non-spool relief valve. Combined with the stability of the flow pattern of grease pipeline with different flow rates given in Table 6, it is concluded that the change of inlet flow rate of grease hardly affects the stable distribution of grease flow pattern in the core pipe. Therefore, it shows that the flow of grease in the core pipe of the spool-less relief valve has been in a steady or slightly fluctuating state, and the change of inlet flow has nothing to do with the diameter of the pipe. At the same time, regarding the above five groups of numerical simulation results of the flow pattern distribution of NLGI1 lithium grease, the flow characteristics of NLGI1 lithium grease in the core tube of the spool-less relief valve are obtained, which is shown by the relative zero velocity movement between the flow layers of grease in the center of the pipeline, that is, the close rest state with zero velocity gradient. It maintains the highest velocity gradient between the flow layers around the circumference of the pipe wall, showing a macroscopic plug flow pattern as a whole.

**Figure 16:**
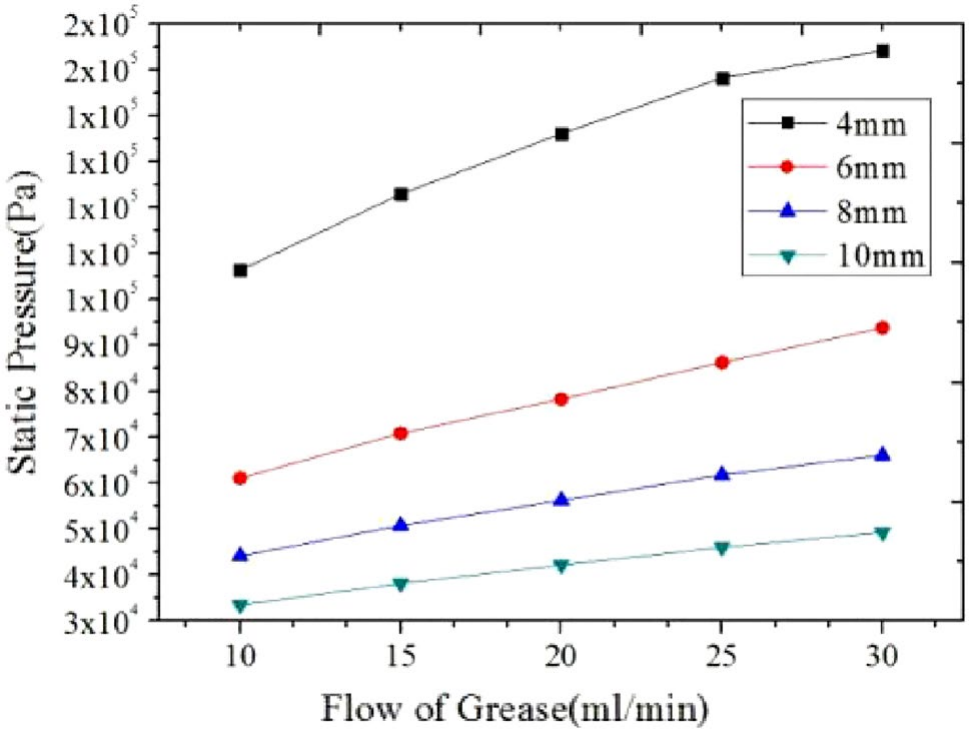
Relationship between pipe flow pressure and flow rate of grease.

Figure 16 mainly shows the correlation characteristic curve between overflow flow and overflow pressure of grease in the core tube of the spool-less relief valve^16^. Analysis of the results of the numerical simulations comparing five sets of simplified models shows that during the five steps of setting the flow rate from 10 to 30 ml/min continuously, the overflow pressure of NLGI1 lithium grease in the core pipe of relief valve increases gradually with the increase of flow rate, and the relationship between flow rate and pressure is linear under the same pipe diameter. In other words, the calibrated lipid flow can ensure the stability of the flow and realize the feasibility of pressure gradient change. In addition, there is also a certain internal relationship between the overflow pressure between the diameters. When the grease keeps the same flow rate, the smaller the diameter of the core pipe of the relief valve is, the greater the corresponding overflow pressure is, and the greater the corresponding pressure gradient between the diameters is. At the same time, combined with the numerical simulation results summarized in Table 7, the five groups of grease flow rate corresponding to the pressure ratio of the pipe diameter change relationship show good consistency; Negligible effect on the pressure ratio between adjacent pipe diameters caused by changes in grease flow. Under the same grease flow rate, the grease overflow pressure ratio of adjacent pipe diameter decreases with the increase of pipe diameter, which shows that the grease overflow pressure value in each pipe diameter maintains the same linear relationship; that is, the grease can show an excellent stable flow.

### Numerical simulation results and analysis of flow pattern distribution

In order to further analyze the distribution law of grease flow pattern in the core pipe, the analysis was carried out in combination with Fluent 15.0 software under certain conditions of setting tube length to explore the morphological distribution pattern of grease circular flow curve for each tube diameter.

**Figure 17:**
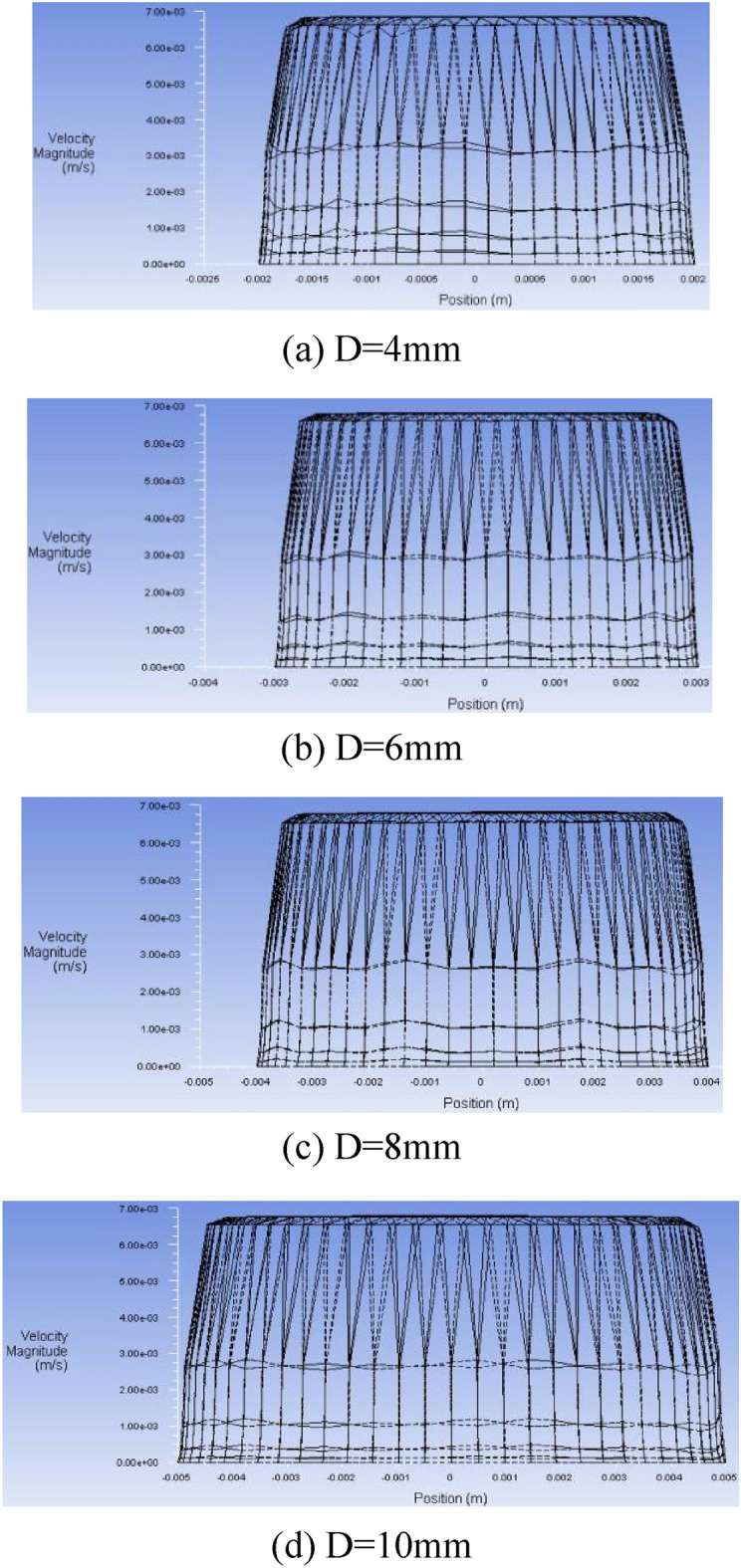
Distribution diagram of flow pattern curve of grease pipe for flow rate 30 ml/min.

Selected 4 mm tube diameter 100 mm tube length of the core round tube, refer to the basic parameters of the boundary conditions in Table 1, the inlet flow rate of 30 ml/min, the outlet pressure is still set to 0 Mpa, through the numerical simulation Fluent 15.0 software calculated grease round tube flow pattern curve distribution law as shown in Fig. 17.

The distribution diagram of the grease flow shape curve in the pipe under the initial condition of the flow of 30 ml/min is obtained by Fluent15.0 fluid analysis software. As shown in Fig. 17, the flow pattern of grease in four kinds of pipe diameter shows obvious plug flow shape characteristics, and the plug flow degree of NLGI1 lithium grease in the pipe is the same, its flow core radius is close to the pipe diameter, and the plug flow degree is very high, this is consistent with the description of the flow pattern of the circular tube flow of grease in the previous theoretical analysis. Because the change law of the flow pattern distribution characteristics of the corresponding grease in the core tube under the other groups of flow is consistent with the numerical simulation cloud image given above, given in Table 8 below, it more intuitively shows the flow morphology characteristics of grease under pipe diameter-flow rate.

**Table 8:** Flow pattern characteristics of grease with different pipe diameters and flow rates.

Pipe diameter (ml/min)	Plug flow pattern			
Pipe diameter (mm):	4	6	8	10
10	Weak fluctuation of plug flow	Weak fluctuation of plug flow	High degree of plug flow	Weak fluctuation of plug flow
15	Weak fluctuation of plug flow	Weak fluctuation of plug flow	High degree of plug flow	Weak fluctuation of plug flow
20	Weak fluctuation of plug flow	High degree of plug flow	High degree of plug flow	Weak fluctuation of plug flow
25	Weak fluctuation of plug flow	High degree of plug flow	High degree of plug flow	Weak fluctuation of plug flow
30	Weak fluctuation of plug flow	Weak fluctuation of plug flow	High degree of plug flow	Weak fluctuation of plug flow

To sum up, the plug flow pattern of grease in the pipeline has nothing to do with the inlet flow rate, pipe diameter, and pipe length set in the channel but is related to its soap fiber structure and semi-solid flow characteristics^17^. By analyzing the flow pattern distribution characteristics of grease in the core pipe of the non-spool relief valve, the regular conclusion can provide a reference for the design and selection of the core pipe of the grease spool-less relief valve.

## Grease valve overflow valve grease spool-free relief valve can check

Based on the analysis and research of the numerical simulation of the flow field in the core component (pipe) of the grease spool-less relief valve, a test rig for the flow in the core pipe of the grease spool-less relief valve is set up to explore the relationship between the flow rate and pressure of the grease in the tube, and further, verify the correctness of the numerical simulation results. By comparing the experimental results with the numerical simulation results, the feasibility and correctness of the theoretical model analysis results are obtained^18^, and the influencing factors and laws are explored. To sum up, the plug flow pattern of grease in the pipeline has nothing to do with the inlet flow rate, pipe diameter, and pipe length set in the channel but is related to its soap fiber structure and semi-solid flow characteristics^17^. By analyzing the flow pattern distribution characteristics of grease in the core pipe of the non-spool relief valve, the regular conclusion can provide a reference for the design and selection of the core pipe of the grease spool-less relief valve.

### Design method of test system

Based on the three basic equations of mass, energy, and momentum conservation^19^ in fluid mechanics, combined with the constitutive equation of NLGI1 lithium grease, the basic theoretical models of flow velocity and shear rate in the core pipe of grease spool-less relief valve are established to analyze and explore the change law of grease in the process of pipe overflow. Through a series of calculations, the flow Eq. (13) and velocity Eq. (12) which accord with the flow of grease in the core pipe of the spool-less relief valve, are derived. From the flow Eq. (13), it is known that the flow rate of grease in the pipeline is affected by many physical parameters, including the shear rheological parameters of NLGI1 lithium grease: shear stress $\tau$, consistency coefficient k, shear-thinning index n, and the pipe diameter R and the pressure gradient $\partial p/\partial z$ along the axial direction of the pipe in the conveying parameters.

Lubricating grease has thermorheological properties, and rheological parameters change with temperature^20^. Therefore, it is necessary to fix the temperature of lubricating grease to carry out the experimental study on the performance of the grease spool-less relief valve. The test maintains a constant temperature of 25 °C water bath, so the shear rheological parameters of nlgi1 lithium grease remain stable. Only the influence of the change of pipe diameter R and pressure gradient $\partial p/\partial z$ in the conveying system on the flow of grease in the pipeline must be considered. While several groups of pipe diameters R in the test are explicitly given, only the pressure gradient $\partial p/\partial z$ parameter is unknown. Under a specific pipe diameter and length, the experiment only needs to measure the flow and pressure value of lubricating grease in the pipeline through the instrument to obtain the variation law between the flow and pressure value. Limited to the experimental conditions in the test process, the experimental research on the performance of grease non-spool relief valve only needs to carry out the experimental verification of the typical indexes; these indicators then prove the feasibility and correctness of the design approach.

### Performance test principle of core round pipe of grease valve device

Based on the analysis of the flow performance of grease in the pipeline based on the flow and pressure, the performance research test platform of grease non-spool overflow valve is built, and the performance test of grease overflow valve under three different parameters of round pipe length, pipe diameter, and grease flow is carried out.

**Figure 18:**
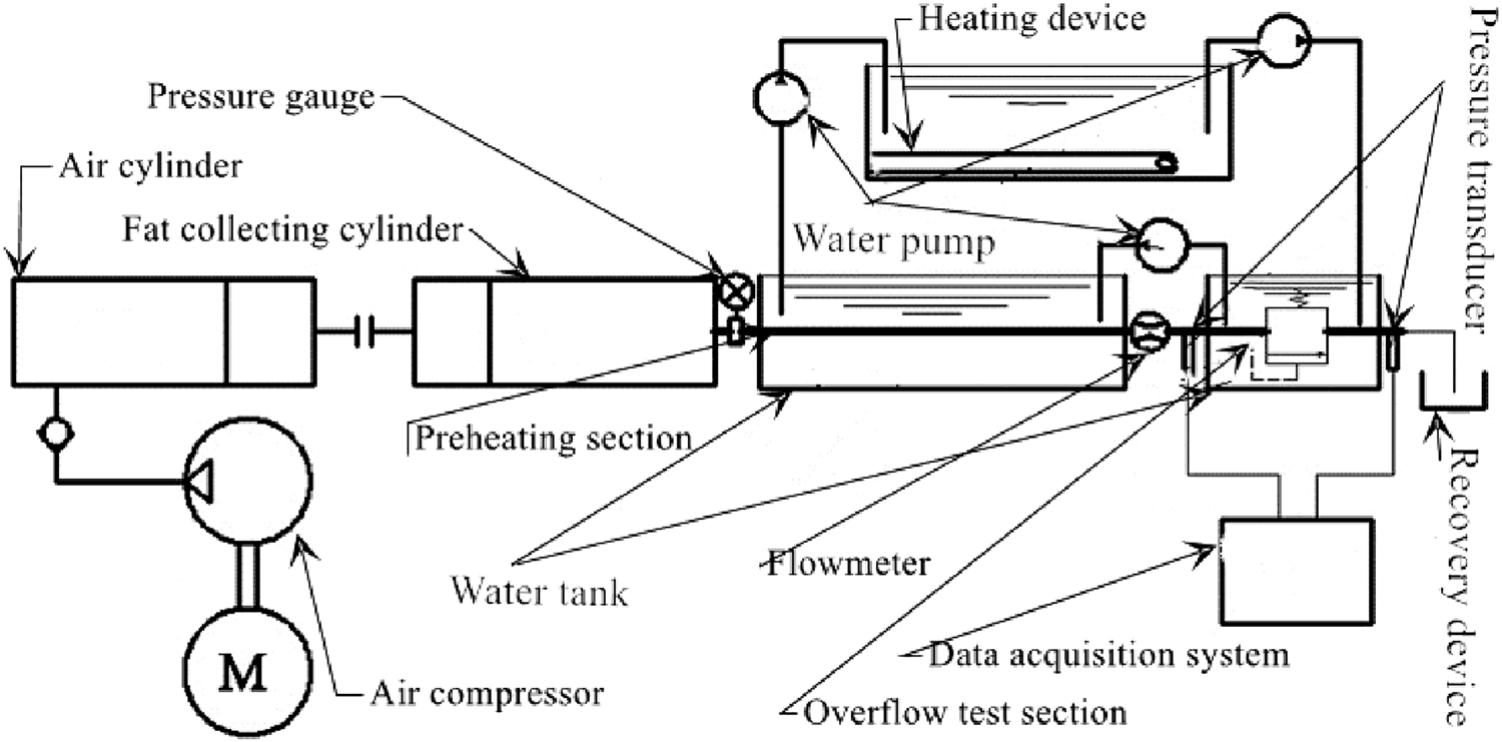
Schematic diagram of working principle of core circular pipe flow test bench of grease non spool overflow valve.

The working principle of the core circular pipe flow test bench of the grease non-spool overflow valve is given in Fig. 18. The test mainly includes the transmission of grease, the thermal insulation treatment of grease, and the collection of flow and pressure in the test pipe section of the grease overflow valve. The test bench uses the air compressor as the transmission power of lubricating grease to promote the flow of lubricating grease in the core circular pipe of the non-spool overflow valve. Because the shear rheological properties of grease are affected by temperature, the temperature of grease is best kept at 25 °C during the design test. Therefore, the electric control water bath heating device is adopted to control the temperature required to examine lubricating grease. The device can effectively avoid the difference in thermal conductivity of lubricating grease in the process of pipeline flow, resulting in the change of shear rheological characteristics of lubricating grease and the deviation of test results. The water heated by the electric control water bath heating device is pumped into two parts through the water pump: the pipeline preheating section and the test section. Both pipelines are placed in the water tank. During the test, the hot water pumped by the water pump first passes through the pipeline of the test section, flows through the preheating section, and finally is transported to the electric heating water tank for heating again. The reverse transmission of hot water can effectively ensure the constant water temperature in the whole heating water circulation process and eliminate the interference of temperature on the test results. In the test stage, pump the grease to the preheating pipe section for sufficient water bath constant temperature heating, and then send it to the pipeline test section to measure grease flow and pipeline pressure. Among them, the elliptical gear flowmeter arranged at the outlet end of the test pipeline is used to measure the grease flow; The pressure sensors are placed at both ends of the inlet and outlet of the test pipeline, and the pressure values at both ends of the test pipeline are obtained through computer data acquisition. Then the relationship between flow and pressure in the test pipeline is obtained.

**Figure 19:**
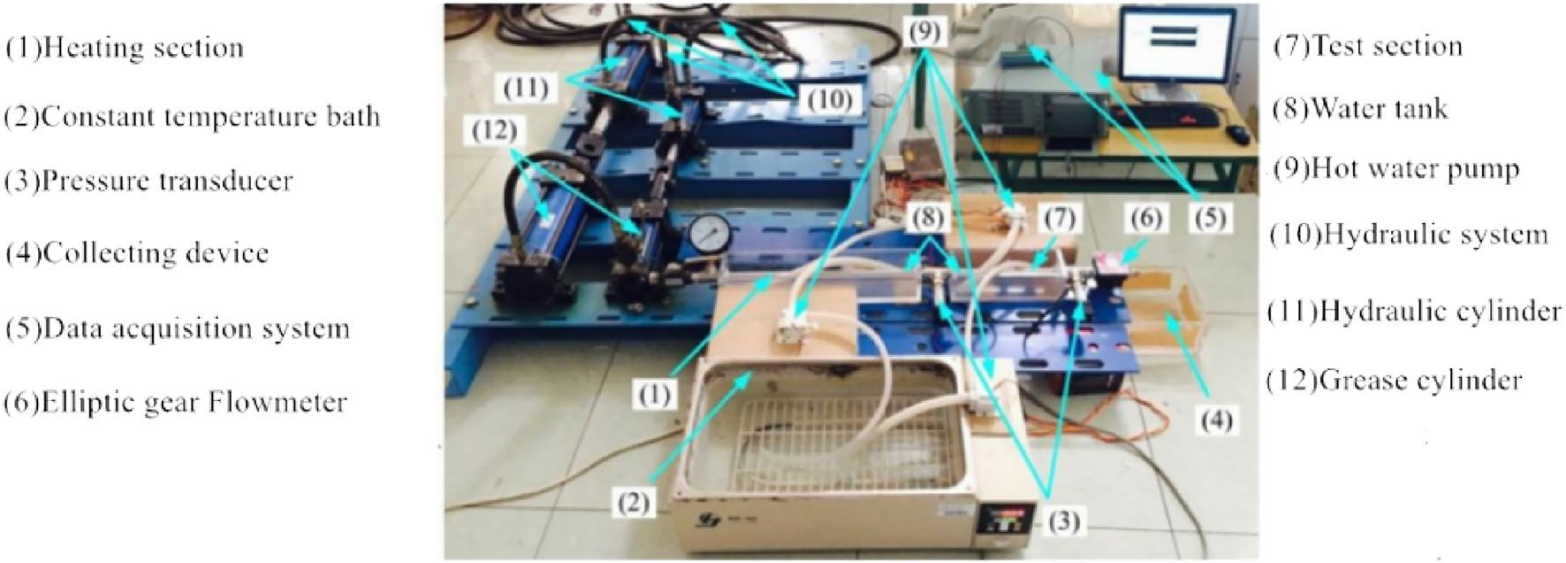
Flow test bench for core circular pipe of grease non spool overflow valve.

According to the working principle of the grease mentioned above spool free overflow valve core circular pipe flow test-bed, build the grease spool free overflow valve core circular pipe flow test-bed shown in Fig. 19. The test bench mainly includes Grease conveying system, electric control water bath heating device, test pipeline, elliptical gear flowmeter, computer data acquisition system, and grease overflow collection device. 0Cr18Ni9 seamless stainless steel pipe is selected as the core round pipe of the non-spool overflow valve in the test. Among them, the inner diameter of the round pipe in the preheating section is 10 mm, the pipe length is 400 mm, the inner diameter of the round pipe in the test section is 10 mm, and the pipe length is 100 mm (3 branch pipes). Firstly, the lubricating grease is transported to the preheating pipe section with the help of the lubricating grease pumping system, and the electric control water bath heating device is used to fully heat the preheating pipeline filled with lubricating grease at a constant temperature until it reaches the temperature required for the test stage. In the test stage of the test bench, the lubricating grease in the preheating pipe section is directly transmitted to the test section. The computer data acquisition system monitors the pressure at both ends of the inlet and outlet of the core pipeline of the valve free overflow valve. By reading the value of the elliptical gear flowmeter, the influence law of the flow rate and resistance of the core circular pipe of the valve free overflow valve is found. The test bench is equipped with several groups of non-spool overflow valve core circular pipes with different pipe diameters, which can further study the effects of pipe diameter, material, and pipe wall roughness on the flow performance of grease circular pipes. The experimental results are compared with the previous theoretical analysis results, the error caused by the simplification of the mathematical model in the process of theoretical research is analyzed, and the error of the theoretical model is corrected to obtain the pressure drop characteristic model in line with the actual working condition of the relief valve.

### Analysis of test results

**Figure 20:**
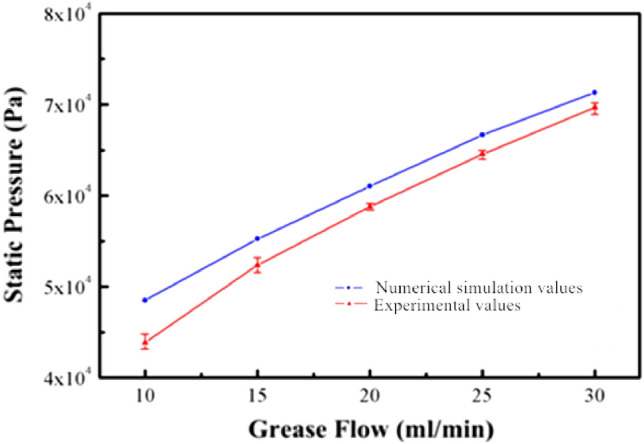
Comparison between numerical simulation value and experimental value of variation law of pressure with flow.

Figure 20 compares the numerical simulation value and the experimental value of the variation law of the pressure with the flow during the flow of the grease round pipe (100 mm long and 100 mm inner diameter)^21^. The graph shows that the experimental values during the experiment were all very concentrated; four measurements were carried out. The average value was taken as the final theoretical value of the circular tube flow of grease; the error bar analysis in the graph shows that the maximum and minimum values are very close to each other. The average value and the experimental results have excellent repeatability.

As can be seen from the figure, the experimental results and the numerical simulation solutions show a good agreement. The pressure value of grease flow in the overflowing core (circular pipe) of the grease non-spool overflow valve increases with flow increase. The experimental results and numerical simulation results show the same trend. This trend is mainly attributed to the fact that the higher the flow rate during grease flow, the more pronounced the change in shear rate during grease flow, which causes an increase in flow resistance. At the same time, the experimental value is always lower than the numerical simulation value, which is mainly because, in the process of numerical simulation of grease circular pipe flow, some simplification and assumptions are made on the grease circular pipe flow process, which leads to a certain error between the experimental and numerical simulation values^22^. Among them, the assumption that there is no slip on the wall of the circular pipe flow in the numerical simulation process is the main reason why the experimental value of the circular pipe flow of grease is lower than the numerical simulation value. There is a wall slip effect in the round pipe flow of grease, and the wall slip effect has an excellent promoting impact on the round pipe flow of grease; as a result, the experimental value of the grease in the experimental testing process is lower than the numerical simulation value of the non-slip hypothesis. In addition, with the increase of the flow rate, the experimental values and numerical simulation values approach each other, mainly because with the rise of the flow rate, the influence of wall slip is weakened, resulting in the effect of the two approaches each other^23^. Therefore, in the initial overflow stage of the grease spool-free relief valve (under minor flow conditions), special attention needs to be paid to the effect of wall slippage on the opening of the relief valve.

## Conclusion

From theoretical analysis, numerical simulation analysis, and experimental research, this paper analyzes and researches the rheological properties of NLGI1 lithium grease and the design and performance of special grease spool-free relief valve with pipeline protection. The conclusions are as follows:

1. Centralized lubrication system conveying grease is different from the hydraulic medium (such as: water, hydraulic oil) conveyed by ordinary hydraulic system, grease shows a higher consistency and is very easy to be blocked during the flow of narrow gaps. In this paper, it is of practical significance and value to study the characteristics of grease with moderate consistency level and develop a grease spool-less relief valve device work that can achieve overflow protection.
2. Design a spool-less relief valve device using a circular tube as the core prototype, based on the resistance of the grease along the tube as the overflow pressure of the valve. Based on the hydrodynamics and the plug flow characteristics of NLGI1 lithium grease, the core pipe flow resistance model of NLGI1 lithium grease is established, and its flow resistance model is analyzed. It is concluded that the variation law of flow resistance and plug flow shape in the core pipe is related to pipe diameter, pipeline resistance, and grease flow rate.
3. Based on the numerical simulation analysis of the simplified model of grease spool-less relief valve under different influence parameters, it is concluded that the overflow pressure of grease flow along the pipeline is positively correlated with the pipe length when the pipe diameter is fixed. In contrast, the pipe length is negatively correlated. The grease overflow capacity follows: the inlet and outlet flow velocity of grease in the core tube of the spool-free relief valve is not affected by pipe diameter and pipe length; the overflow pressure of grease is positively correlated with its overflow flow, and the same pipe diameter, the pressure and flow rate maintain a nearly linear correlation; with the same flow rate, there is a positive correlation between the diameter of the core pipe and the overflow pressure of grease. The flow pattern of grease follows: the flow pattern of grease in the pipeline shows a high shear rate near the pipe wall, and the flow core in the central section of the pipeline maintains a relatively static zero shear rate. It accords with the plug flow pattern established in the theoretical model of grease circular pipe flow.
4. The numerical simulation results agree with the experimental results. The pressure value of grease flow in the overflowing core (pipe) of the grease spool-less relief valve increases with the flow rate increase. The experimental results and numerical simulation results show the same trend. However, there is a wall slip effect in the grease pipe flow, so the numerical simulation results of the pressure change with the flow rate are higher than the experimental values.
5. Grease in the core circular tube flow process, its interaction with the inner wall surface, there is a certain influence, in view of this influence effect is weak and complex, not considered grease in the pipeline formed in the wall slip effect. If this influence factor is taken into account, the grease without spool relief valve performance can be analyzed more comprehensively and effectively.

## Data Availability

The data used to support the findings of this study are included within the article.
